# A new nematode species, *Tanqua siamensis* sp. nov. (Nematoda: Gnathostomatidae) in the rainbow water snake, *Enhydris enhydris*, from Thailand

**DOI:** 10.1017/S0031182024000908

**Published:** 2024-07

**Authors:** Vachirapong Charoennitiwat, Urusa Thaenkham, Supakit Tongpon, Kittipong Chaisiri, Panithi Laoungbua, Tanapong Tawan, Tapanee Kanjanapruthipong, Sumate Ampawong, Abigail Hui En Chan, Napat Ratnarathorn

**Affiliations:** 1 Department of Helminthology, Faculty of Tropical Medicine, Mahidol University, Bangkok, Thailand; 2 Animal Systematics & Molecular Ecology Laboratory and Applied Animal Science Laboratory, Department of Biology, Faculty of Science, Mahidol University, Bangkok, Thailand; 3 Snake Farm, Queen Saovabha Memorial Institute, The Thai Red Cross Society, Bangkok, Thailand; 4 Department of Tropical Pathology, Faculty of Tropical Medicine, Mahidol University, Bangkok, Thailand

**Keywords:** molecular identification, morphology, rainbow water snake, snake parasite, *Tanqua*, Thailand

## Abstract

The genus *Tanqua* Blanchard, 1904, infests reptiles, particularly those inhabiting aquatic environments. This study examined a population of rainbow water snakes, *Enhydris enhydris* (Schneider, 1799), collected from southern Thailand. Adult nematodes consistent with *Tanqua* were found in the stomach. Various morphometric, meristic and qualitative morphological variables, including size, ratios, distances, cephalic appearance, the number of caudal papillae and other features, serve to distinguish the specimens from other species within the genus. In particular, *Tanqua anomala* and *Tanqua diadema*, which closely resemble our *Tanqua* specimens, can be differentiated by key diagnostic characteristics such as a retractable head, the distance from the anterior end to the cervical sac, the relative positions of caudal papillae and excretory pore, and the length of the uterus. Molecular analysis (*COI* and 18s rRNA genes) confirmed its status as a species of *Tanqua*, genetically distinct from *Tanqua tiara*, and matching the genetic sequence found in larvae of *Tanqua* sp. from a snakehead fish species from Bangladesh. *Tanqua siamensis* sp. nov. is described, supported by morphological traits, microscopic illustrations and genetic information. This study reports the first evidence of a caudal papillary pair in females. This species causes significant lesions on the stomach wall of the snake host, raising possible issues for snakes held in captivity regarding food hygiene and parasite protection.

## Graphical abstract

**Figure:**
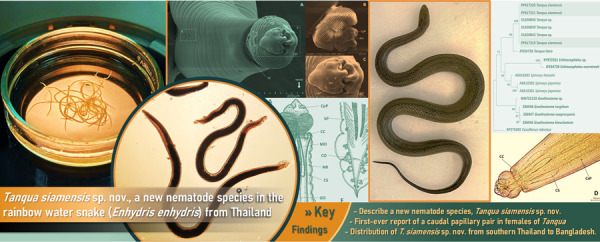

## Introduction

Nematodes of the genus *Tanqua* Blanchard, 1904, infests the stomach and intestine of reptiles, particularly snakes inhabiting aquatic habitats (Baylis, 1916; Dewi *et al*., 2008; Agustin *et al*., 2017). Species of the genus display a stout body, a cephalic bulb with a posteriorly encircled cuticular collar, a thick cuticle with transverse striations, 2 transversely striated pseudolabia projected anteriorly, interdigitating tooth-like lips, a long oesophagus that gradually increases in diameter posteriorly and is clearly separated from the intestine, and a tapering tail with ventral papillary pairs (Baylis, 1916; Baylis and Lane, 1920).

The type-species, *Tanqua tiara* (von Linstow, 1879), initially described as a species of *Ascaris*, underwent reclassification, including genetic characterization conducted by Laetsch *et al*. (2012) and the latest redescription was by Sou (2020), rendering it the most studied member of the genus (e.g. Gibbons and Keymer, 1991; Agustin *et al*., 2017). Following *T. tiara*, *Tanqua anomala* (von Linstow, 1904) and *Tanqua diadema* (von Linstow, 1904) were examined and confirmed as valid species by Baylis (1916) and Baylis and Lane (1920). *Tanqua ophidis* Johnston & Mawson, 1948, was described in both the common keelback snake, *Tropidonophis mairii*, and a file snake species, *Acrochordus* sp., in Australia (Johnston and Mawson, 1948; Kagei and Shogaki, 1977). However, later research proposed that *T. ophidis* is synonymous with *T. anomala* (Dewi *et al*., 2008). Similarly, *Tanqua sindensis* Farooq *et al*., 1979, was also considered synonymous with *T. anomala*, as reviewed by Bilqees (1980). In the mid-late 20th century, several additional species of *Tanqua* were described. These are *Tanqua occlusa* Schuurmans-Stekhoven, 1943, described in the Smith's African water snake, *Grayia smithii*; *Tanqua gigantica* Kung, 1948, described in the reticulated python, *Malayopython reticulatus*, and the king cobra, *Ophiophagus hannah*; *Tanqua bainae* Ghadirian, 1968, described in the Madagascar tree boa, *Sanzinia madagascariensis*; and *Tanqua geoclemydis* Wang *et al*. 1979, described in the Chinese pond turtle, *Mauremys reevesii*. No further species have been discovered since.

The rainbow water snake, *Enhydris enhydris* (Schneider, 1799), eats fish and occasionally preys on small amphibians and reptiles (Vattakaven *et al*., 2016). As predators, they can significantly impact parasite transmission by acting as reservoirs that facilitate the completion of a parasite's life cycle, especially when they consume infected hosts (Vattakaven *et al*., 2016; Lopez and Duffy, 2021). Using both morphology and molecular methods, the nematodes infecting *E. enhydris* were identified. Genetic data revealed a match with *Tanqua* sp. (*sensu* Williams *et al*., 2022), a larval stage previously observed in the spotted snakehead fish, *Channa punctata*. Genetic markers employed for the molecular identification of *Tanqua* include the nuclear 18S and 28S ribosomal RNA (rRNA) genes. However, only 1 sequence has been associated with an identified species (*T. tiara*). Here, we describe a new nematode species, namely *Tanqua siamensis* sp. nov., supported by microscopic illustrations, morphological characteristics and genetic information.

## Materials and methods

### Host and parasite specimen preparation

Rainbow water snakes, *E. enhydris*, captured by local villagers and rescuers from Nakhon Si Thammarat Province and adjacent provinces in the southern part of Thailand, were delivered to the Snake Farm (SF), Queen Saovabha Memorial Institute (QSMI) in Bangkok, Thailand. Between 2020 and 2023, twelve specimens that perished during the quarantine stage were preserved at −20°C before undergoing dissection to explore helminths, adhering to reptile necropsy protocols (Terrell and Stacy, 2007). Prior to dissection, the snakes underwent scrutiny based on Cox *et al*. (2012) criteria. Various meristic and measurement variables, including weight, snout-vent length, tail length, scale numbers at different positions, gender and body pattern, were examined to confirm their species and gather host data.

After the dissection, organs, particularly the stomach, were isolated from each snake and placed in a petri dish filled with tap water. The organs were opened and carefully examined under stereomicroscopes (Olympus SZ30 and SZ51, Japan). Micro dissecting needles and precision probes were employed to extract all parasites, particularly *Tanqua* found in the stomach. These parasites were then transferred to a small petri dish filled with 0.85% normal saline and subsequently preserved in 70% ethanol in 1.5 mL sampling tubes. The number of parasites obtained per organ per snake was documented. Tubes containing parasites and the remaining parts of the snake specimens were stored in −20°C freezers at the Department of Helminthology, Faculty of Tropical Medicine, and the Department of Biology, Faculty of Science, Mahidol University, respectively.

### Morphological study

For morphological studies, 32 male and 28 female complete helminth specimens were chosen from the preserved 70% ethanol stock for the creation of permanent slides. A subset of 6 specimens (3 males and females), in excellent body condition, were specifically selected as holotype, allotype and paratypes. Each specimen was stained in acetocarmine and dehydrated by sequential immersion in 70, 80, 90, 95% and concentrated ethanol for 45 min at each step. For neutralization and clearing, the specimens were then submerged in a 1:1 ethanol: xylene solution for 45 min, followed by a brief immersion in xylene. Subsequently, each specimen was placed in a few drops of mounting medium (Permount™) on a glass slide, covered with a coverslip, allowed to cool for a few minutes and incubated at 60°C for several days. The remaining specimens (*n* = 53) were mounted using lactophenol.

A comprehensive examination was conducted using an inverted microscope (Zeiss, Primovert, Germany) equipped with a Zeiss Axiocam and ZEN2 blue edition software. All measurements were recorded in millimetres (mm). Taxonomic keys for the identification of *Tanqua* species and the morphological features for species identification were derived from Baylis (1916), Dewi *et al*. (2008), Agustin *et al*. (2017) and Sou (2020). Illustrations were generated through drawings using a light microscope with a camera lucida (Leitz, Wetzlar, Germany).

For scanning electron microscope (SEM) analysis, 3 male and female specimens were selected from the preserved 70% ethanol stock. Initially, these specimens were immersed in a solution containing 2.5% glutaraldehyde in a 0.1 M sucrose phosphate buffer (SPB) for primary fixation. Subsequently, a secondary fixation step was performed using a 1% osmium tetroxide solution in the same 0.1 M SPB. Following this, the specimens were dehydrated with ethanol and dried using a critical point drying device (CPD300 auto, Leica, Wetzlar, Germany). A fine coating of gold was applied using a sputter coater (Q150R PLUS, Quorum, East Sussex, England). The specimen preparation was conducted at the Department of Tropical Pathology, Faculty of Tropical Medicine, Mahidol University. Finally, these prepared specimens were examined under the SEM (Hitachi, SU8010, Japan). The SEM analysis took place at the Faculty of Science (Phaya Thai), Mahidol University.

To examine morphological variation among all 60 helminth specimens (32 males and 28 females), 17 morphological characteristics shared by both genders were analysed. These were body length, maximum body width, cephalic bulb diameter, pseudolabial width, head length, distance from anterior to oesophagus end, maximum oesophagus width, muscular oesophagus length, muscular oesophagus width, glandular oesophagus length, glandular oesophagus width, distance from anterior to cervical sac, distance from anterior to nerve ring, distance from anterior to excretory pore, distance from anterior to cervical papillae, cuticle thickness and tail length (see Table 1). To assess the morphological variation of the specimens between hosts, gender morphologies were additionally employed. This included the number of caudal papillary pairs and spicule length for males (accounting for a total of 19 characters), and vulva to posterior end, egg width and egg length for females (accounting for a total of 20 characters) (Table 1). The multivariable data matrices were imported into principal component analysis (PCA) using PAST version 4.06b software (Hammer *et al*., 2001). A correlation matrix model was employed to generate 2-dimensional scatter plots showing the percentage variances.

**Table 1.** Information and measurement characters for *T. tiara*, *T. anomala* and *T. siamensis* sp. nov

Examined characters		*T. siamensis* sp. nov. (this study)	*T. anomala* (Baylis, 1916; Dewi *et al*., 2008)	*T. tiara* (Sou, 2020)	*T. tiara* (Agustin *et al*., 2017)
Host (host taxon)		*Enhydris enhydris* (Serpentes)	*Acrochordus javanicus* (Serpentes)	*Varanus flavescens* (Lizard)	*Varunus salvator* (Lizard)
Side of infection		Stomach	Gastrointestinal tract	Stomach	Stomach
Locality		South Thailand	South Sumatra, Indonesia	West Bengal, India	East Java, Indonesia
Body length	♂	17.00–40.33 (x̄ = 26.39)	25.0–39.5 (x̄ = 35.7)	13.50–39.00	9.4–32.0 (x̄ = 18.6)
	♀	15.33–41.17 (x̄ = 27.73)	33.6–43.9 (x̄ = 37.4)	20.00–45.46	6.8–22.0 (x̄ = 12.2)
Maximum body width	♂	0.51–1.12 (x̄ = 0.85)	0.49–0.89 (x̄ = 0.66)	0.19–1.10	0.26–1.77 (x̄ = 0.66)
	♀	0.56–1.56 (x̄ = 0.94)	0.67–0.95 (x̄ = 0.84)	0.53–1.51	0.14–2.33 (x̄ = 0.60)
Cephalic bulb diameter	♂	0.19–0.38 (x̄ = 0.28)	0.17–0.27 (x̄ = 0.19)	–	0.17–0.32 (x̄ = 0.25)
	♀	0.22–0.44 (x̄ = 0.30)	0.19–0.29 (x̄ = 0.24)	–	0.15–0.34 (x̄ = 0.21)
Number of cephalic swellings		2	2	4	–
Pseudolabial width	♂	0.12–0.24 (x̄ = 0.18)	0.13–0.18 (x̄ = 0.16)	–	
	♀	0.10–0.30 (x̄ = 0.18)	0.15–0.21 (x̄ = 0.17)	–	
Head length	♂	0.21–0.40 (x̄ = 0.30)	–	–	0.23–0.33 (x̄ = 0.26)
	♀	0.22–0.46 (x̄ = 0.30)	–	–	0.10–0.28 (x̄ = 0.15)
Distance from the anterior end to oesophagus end	♂	1.53–4.72 (x̄ = 3.49)	3.22–5.80 (x̄ = 4.03)	3.00–6.51	2.80–5.70 (x̄ = 4.20)
	♀	2.55–5.39 (x̄ = 3.59)	3.45–4.24 (x̄ = 3.77)	4.00–9.02	2.30–4.58 (x̄ = 3.18)
Maximum oesophagus width	♂	0.29–0.54 (x̄ = 0.42)	0.28–0.39 (x̄ = 0.40)	0.40–0.42	–
	♀	0.21–0.67 (x̄ = 0.42)	0.28–0.52 (x̄ = 0.38)	0.15–0.46	–
Muscular oesophagus length	♂	0.22–0.49 (x̄ = 0.35)	–	0.63–0.65	–
	♀	0.23–0.69 (x̄ = 0.38)	–	0.67–0.68	–
Muscular oesophagus width	♂	0.08–0.30 (x̄ = 0.19)	–	0.12–0.14	–
	♀	0.09–0.34 (x̄ = 0.21)	–	0.15–0.16	–
Glandular oesophagus length	♂	1.28–4.17 (x̄ = 3.02)	–	3.40–3.64	–
	♀	2.21–4.61 (x̄ = 3.13)	–	4.81–4.86	–
Glandular oesophagus width	♂	0.29–0.54 (x̄ = 0.42)	–	0.40–0.42	–
	♀	0.21–0.67 (x̄ = 0.42)	–	0.44–0.46	–
Distance from the anterior end to cervical sac	♂	0.12–0.20 (x̄ = 0.16)	0.57–0.68 (x̄ = 0.61)^a^	–	0.22–0.81 (x̄ = 0.37)
	♀	0.10–0.22 (x̄ = 0.17)	0.68–0.81 (x̄ = 0.74)^a^	–	0.18–0.88 (x̄ = 0.33)
Number of cervical sacs		4	4	–	4
Distance from the anterior end to nerve ring	♂	0.23–0.50 (x̄ = 0.37)	0.41–0.60 (x̄ = 0.49)	0.45–0.93	–
	♀	0.21–0.55 (x̄ = 0.36)	0.47–0.62 (x̄ = 0.55)	0.48–0.75	–
Distance from the anterior end to excretory pore	♂	0.35–0.66 (x̄ = 0.53)	0.75–0.96 (x̄ = 0.85)	0.72–1.21	–
	♀	0.34–0.73 (x̄ = 0.54)	0.90–1.23 (x̄ = 1.09)	0.81–1.08	–
Distance from the anterior end to cervical papillae	♂	0.39–0.74 (x̄ = 0.59)	0.63–0.79 (x̄ = 0.73)	0.70–1.37	–
	♀	0.40–0.81 (x̄ = 0.59)	0.69–0.85 (x̄ = 0.81)	0.89–1.14	–
Cuticle thickness	♂	0.02–0.05 (x̄ = 0.03)	–	–	0.008–0.036 (x̄ = 0.022)
	♀	0.02–0.05 (x̄ = 0.03)	–	–	0.007–0.031 (x̄ = 0.023)
Tail length	♂	0.23–0.83 (x̄ = 0.56)	0.35–0.49 (x̄ = 0.42)	0.30–0.33	0.13–0.42 (x̄ = 0.28)
	♀	0.59–1.37 (x̄ = 0.94)	0.73–1.11 (x̄ = 0.95)	0.39–0.48	0.12–0.28 (x̄ = 0.19)
Number of caudal papillary pairs	♂	7 or 8: 3 preanal, 1 paranal, 3 or 4 postanal	8: 3 preanal, 1 paranal, 4 postanal	8: 3 preanal, 1 paranal, 4 postanal	–
	♀	1 postanal	–	–	–
Spicule length		1.09–1.87 (x̄ = 1.50)	0.75–1.33 (x̄ = 0.88)	0.77–1.91	0.30–1.10 (x̄ = 0.63)
Vulva from the posterior end		6.68–16.56 (x̄ = 10.59)	7.64–12.27 (x̄ = 10.04)	4.50–7.69	3.02–3.61 (x̄ = 3.31)
Uterus length		Very long (2/5 to half of body length)	Very short	–	13.10–21.40 (x̄ = 17.25)
Number of uterine branches		2	2	–	–
Egg size (W × L)		0.036–0.059 (x̄ = 0.042) × 0.046–0.076 (x̄ = 0.056)	0.0376 × 0.050	–	0.030–0.049 × 0.040–0.052

Diagnostic characters for the new species are indicated in bold type. All measurements in millimetre (mm).

a From the level of anterior margin of cervical collar.

### Molecular and phylogenetic study

For DNA extraction, 5 specimens were homogenized and processed using DNeasy Blood & Tissue Kit (Qiagen, Germany) following the manufacturer's instructions. The genomic DNA extracted was eluted with 30 μL of nuclease-free water and quantified using spectrophotometry.

The amplification targeted a partial sequence of a mitochondrial gene: cytochrome c oxidase subunit I (*COI*) and a nuclear gene: 18S ribosomal RNA (18S rRNA). These gene loci, known for molecular identification and revealing genetic diversity within nematode species, were selected based on previous studies (Tokiwa *et al*., 2012; Eamsobhana *et al*., 2015; Chan *et al*., 2020; Thaenkham *et al*., 2022). The following primers were employed: JB3 5′-TTTTTTGGGC ATCCTGAGGTTTAT-3′ and JB4.5 5′-TAAAGAAAGAACATAATGAAAATG-3′ for *COI*, and 1096F 5′-GGTAATTCTGGAGCTAATAC-3′ and 1916R 5′-TTTACGGTCAGAACTAG GG-3′ for 18S rRNA. The resulting amplicon lengths for *COI* and 18S rRNA were 446 and 800 bp, respectively.

Polymerase chain reaction (PCR) reactions were conducted using a T100^Tm^ thermocycler from Bio-Rad. The reaction mixture had a final volume of 30 μL, including 15 μL of 2X i-Taq master mix (Biotechnology, Gyeonggi, South Korea), 10 μm of each primer and 1 ng μL^−1^ of DNA. Thermocycling profiles varied for different gene targets, following established protocols (Holterman *et al*., 2006; Charoennitiwat *et al*., 2023). PCR amplicons were visualized on a 1% agarose gel stained with SYBR Safe (Thermo Fisher Scientific, Waltham, USA). The PCR products from 3 specimens were sequenced using Barcode Taq sequencing (Celemics, Seoul, South Korea). Nucleotide sequences from this study were submitted to the NCBI database with the accession numbers PP444683–84 for *COI* and PP417319–21 for 18S rRNA.

The partial sequences of the 2 target genes were verified through manual inspection of electropherograms using BioEdit version 7.2.5, and the sequences were aligned using ClustalX 2.1. Phylogenetic analysis was performed using maximum likelihood (ML) in MEGA-X with the best-fit nucleotide substitution model and 1000 bootstrap replicates. The nucleotide substitution models used were Tamura-Nei (TN93) with a gamma distribution (+G) for *COI* and Kimura 2-parameter (K2) with a gamma distribution (+G) for 18S rRNA (Hall, 1999; Thompson *et al*., 2002; Tamura *et al*., 2013).

## Results

### Taxonomy

#### Phylum: Nematoda Diesing, 1861 Class: Chromadorea Inglis, 1983 Order: Rhabditida Chitwood, 1933 Family: Gnathostomatidae Railliet, 1895 Genus: *Tanqua* Blanchard, 1904 Species: *Tanqua siamensis* sp. nov. Charoennitiwat *et al*., 2024 (Table 1, Figs 1–3)

**Type-host**: *Enhydris enhydris* (Schneider, 1799)

**Figure 1.**
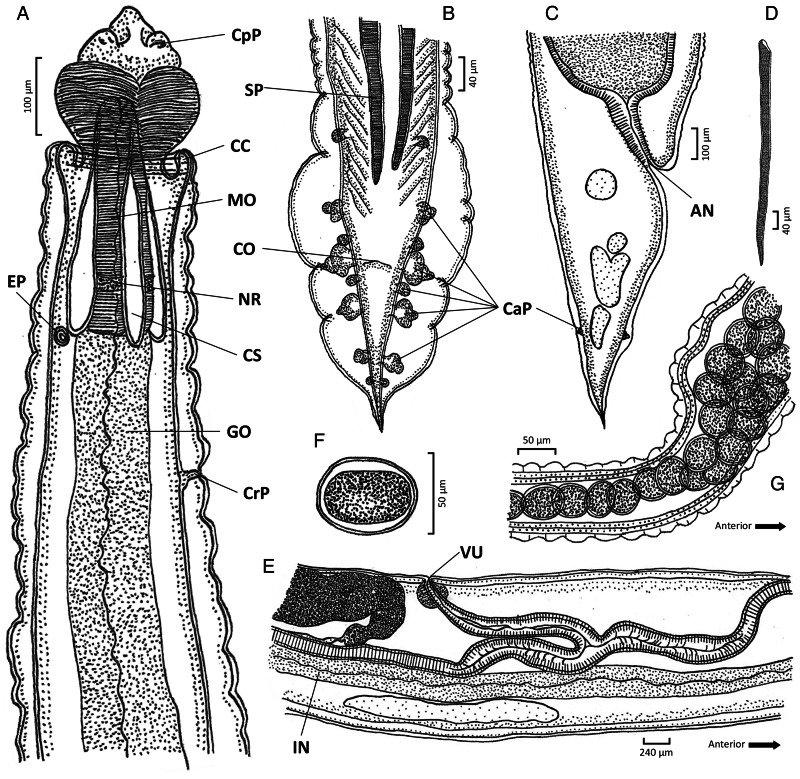
*Tanqua siamensis* sp. nov. of sample IDs: SN064TM01 (♂ paratype) and SN032TF02 (♀ allotype): (A) anterior end of male, lateral view; (B) posterior end of male, ventral view; (C) posterior end of female, lateral view; (D) a spicule of male; (F) eggs in uterus; and (E) reproductive structures of female, lateral view. AN, anus; CaP, caudal papillae; CC, cuticular collar; CO, cloaca; CpP, cephalic papillae; CrP, cervical papillae; CS, cervical sac; EP, excretory pore; GO, glandular oesophagus; IN, intestine; MO, muscular oesophagus; NR, nerve ring; SP, spicule; VU, vulva.

**Figure 2.**
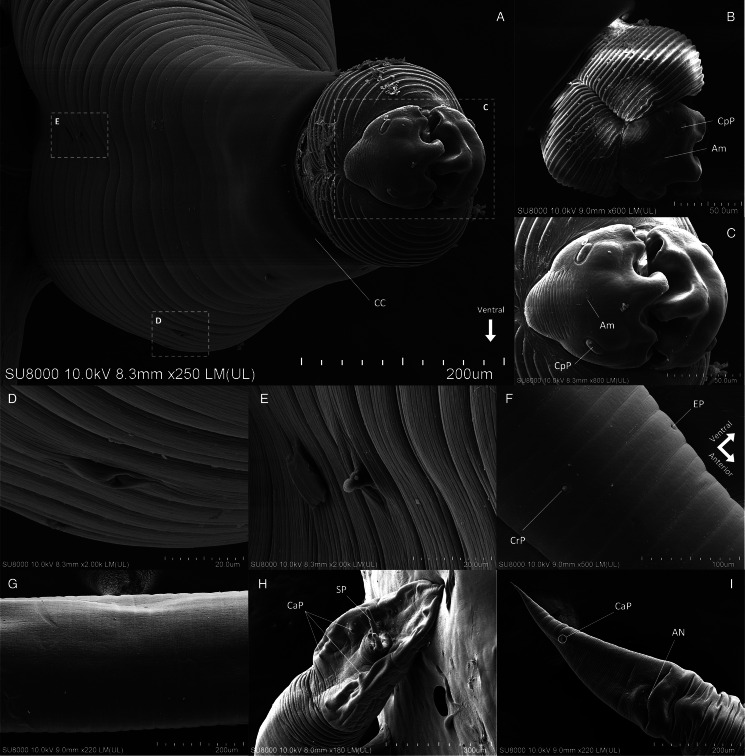
Scanning electron micrograph of *Tanqua siamensis* sp. nov.: (A) anterior region, anterior view; (B) cephalic bulb, lateral view; (C) pseudolabia, anterior view, extended from Fig. A; (D) excretory pore, extended from Fig. A; (E) cervical papilla, extended from Fig. A; (F) order sequence of cervical papilla and excretory pore, lateral view; (G) body wall with transverse striations, lateral view; (H) posterior end of male, ventral view; and (I) posterior end of female, ventral view. Am, amphid; AN, anus; CaP, caudal papillae; CC, cuticular collar; CpP, cephalic papillae; CrP, cervical papillae; EP, excretory pore; SP, spicule.

**Figure 3.**
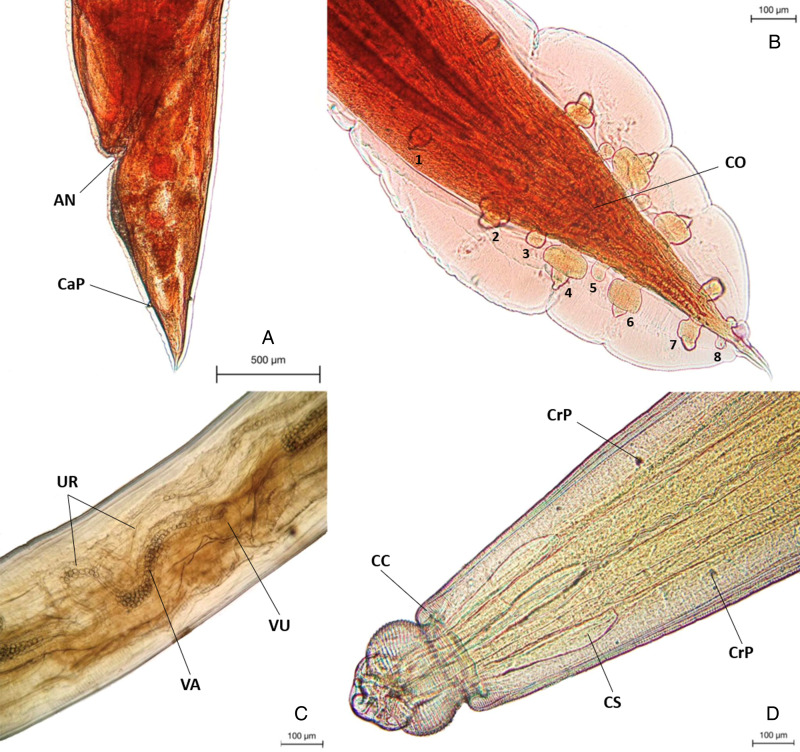
Permanent slides (acetocarmine dye, A and B) and semi-permanent slides (lactophenol, C and D) of *Tanqua siamensis* sp. nov.: (A) posterior region of female, lateral view; (B) posterior region of male, ventral view; (C) reproductive structures of female, ventral view; and (D) anterior region of male, dorsal view. 1‒8, pairs of caudal papillae; AN, anus; CaP, caudal papillae; CC, cuticular collar; CO, cloaca; CrP, cervical papillae; CS, cervical sac; UR, uteri; VA, vagina; VU, vulva.

**Type-locality**: Aquatic areas including lakes, ponds, swamps and paddy fields in Nakhon Si Thammarat (e.g. Pak Phraek, Thung Song district) and adjacent provinces in the southern part of Thailand (e.g. Songkhla lake). Specific coordinates of each host were not recorded.

**Collection date**: 10th November 2020 to 26th August 2023.

**Site of infection**: Stomach (the end of oesophagus and the beginning of small intestine in cases of high intensity)

**Parasite intensity**: 8–52 worms, mean approximately 23

**ZooBank LSID**: urn:lsid:zoobank.org:pub:AF6F528F-3F8A-4060-AF99-733915C59174

**Etymology**: The specific epithet ‘*siamensis*’ indicates that the nematode species is found in Thailand. We propose the colloquial English name for this nematode as the ‘stomach roundworm’ and the Thai name as ‘พยาธิกระเพาะงูสยาม’ (Phayat Krapho Ngu Siam).

### General description

Body elongated with head and tail narrow. Cephalic bulb at anterior end regular, unarmed, with even transverse striations (approximately 23 rows of exposure from cuticular collar, Fig. 2B), and divided by longitudinal grooves into 2 submedian swellings positioned dorsally and ventrally (Figs 1A, 2A, B and 3D). Cuticular collar posterior to cephalic bulb (Figs 1A, 2A and 3D). Two thick pseudolabia project anteriorly, and medial surfaces deeply furrowed, slightly asymmetric (Figs 2A‒C). Intervening ridges appear as 5 blunt, tooth-like features, with lateral ridges smaller than medial ones (Fig. 2C). Projections on each pseudolabium interdigitate with one another (Figs 2A, C). Two sessile cephalic papillae present on external surface of each pseudolabium, featuring cordiform lateral prominences with minute amphid in between (Figs 1A and 2B, C). Oesophagus long, simple, gradually increases in diameter posteriorly (Figs 1A and 3D). Body wall smooth with fine transverse striations (Fig. 2D–G). Excretory pore on medial ventral side (Figs 1A and 2A, D, F), anterior to cervical papillae (Figs 1A and 2A, F). Cervical papillae digitiform on each lateral side (Figs 1A and 2A, F, D), spherical at base, taper abruptly (Fig. 2E). Four cervical sacs extend posteriorly from ballonets (Figs 1A and 3D).

Males (holotype, 2 paratypes and 29 voucher specimens): Body length 17–40.33 with maximum width 0.50–1.12. Cephalic bulb width 0.19–0.38; pseudolabial width 0.12–0.24. Oesophagus 1.52–4.71 long (7.7–20.3% of body length), with maximum width 0.29–0.54. Muscular oesophagus 0.22–0.49 long, with maximum width 0.08–0.30. Glandular oesophagus 1.28–4.17 long, with maximum width 0.35–0.66. Four cervical sacs 0.12–0.20 long, extend from anterior end, nerve ring 0.22–0.50 from anterior end, excretory pore, 0.35–0.66 from anterior end. Cervical papillae 0.39–0.74 from anterior end. Two equal and similar cuticular spicules 1.09–1.87 in length (4–8% of body length), curved ventrally, tubular, with pitted surface, without alae (Figs 1B, D and 2H). Tail tapering to point, 0.23–0.83 long, with well-developed caudal alae extending from anterior to cloaca to tip of tail (Figs 1B, 2H and 3B). Usually 8, sometimes 7, pairs of sessile caudal papillae present, situated ventrolaterally; 2/3 pairs preanal, 1 pair paranal and 4 pairs of diminishing size postanal (Figs 1B, 2H and 3B). First, fourth and sixth pairs from posterior end small, third and fifth pairs of caudal papillae from posterior end extend to alae (Figs 1B and 3B).

Gravid females (allotype, 2 paratypes and 25 voucher specimens): Body length 15.33–41.17, maximum width 0.56–1.56. Cephalic bulb width 0.22–0.44, pseudolabial width 0.10–0.30. Oesophagus 2.55–5.39 long (8.6–20.7% of body length), maximum width 0.21–0.67. Muscular oesophagus 0.23–0.69 long, with maximum width 0.09–0.34. Glandular oesophagus 2.21–4.61 long, with maximum width 0.21–0.67. Four cervical sacs 0.10–0.22 long, extend from anterior end. Nerve ring and excretory pore 0.21–0.55 and 0.34–0.73 from anterior end, respectively. Cervical papillae 0.40–0.81 long from anterior end. Vulva in posterior region of body, 6.68–16.56 from posterior end. Vulva present, 2 directly opposed uterine branches (didelphic) very long, two-fifths to half of body length (Figs 1E and 3C). Tail long and tapering, 0.59–1.37 in length (Figs 1C, 2I and 3A). A pair of caudal papillae located near tail end, with each positioned slightly laterally on both dorsal and ventral sides (Figs 1C, 2I and 3A). Eggs 0.04–0.06 × 0.05–0.08, oval, thin-shelled, ornamented with fine granulations (Fig 1F‒G).

### Type materials

Holotype: Mature male deposited at the Mahidol University Museum of Natural History (Voucher no.: MUMNH-NEM0027; specimen code: SN071TM01) was collected by Vachirapong Charoennitiwat and his team, on 26th August 2023, in the stomach of a rainbow water snake, *Endydris endydris* (IDs: SN071 for this project; AAS077 [CO-Ee-046] for the Applied Animal Science laboratory's catalogue), at the Department of Helminthology, Faculty of Tropical Medicine, Mahidol University. Measurements of the holotype are available in Table S2. Eight pairs of sessile caudal papillae present; 3 pairs preanal, 1 pair paranal and 4 pairs of diminishing size postanal. First, fourth and sixth pairs from posterior end small, third and fifth pairs of caudal papillae from posterior end extend to alae. Other descriptive characters are consistent with the general description.

Allotype: Gravid female deposited at the Mahidol University Museum of Natural History (Voucher no.: MUMNH-NEM0028; specimen code: SN032TF02) was collected by Vachirapong Charoennitiwat and his team, on 21st June 2022, in the stomach of a rainbow water snake, *E. endydris* (IDs: SN032 for this project; AAS037 [CO-Ee-013] for the Applied Animal Science laboratory's catalogue), at the Department of Helminthology, Faculty of Tropical Medicine, Mahidol University. Measurements of the allotype are available in Table S2. Vulva didelphic (Fig. 1E), 1 pair of caudal papillae with each papilla situated on dorsal and ventral sides, laterally to midlines (Figs 1C, 2I and 3A). Eggs oval, thin-shelled, ornamented with fine granulations (Fig. 1F‒G). Other descriptive characters are consistent with the general description.

Paratypes (1–4) all from the stomach of *E. endydris*. Two males – Voucher no.: MUMNH-NEM0029 and MUMNH-NEM0030; specimen code: SN062TM01 and SN068TM01, respectively – were collected from snake IDs SN064 and SN068 (or AAS070 [CO-Ee-039] and AAS074 [CO-Ee-043]) on 26th August 2023. Two females – Voucher no.: MUMNH-NEM0031 and MUMNH-NEM0032; specimen code: SN032TF01 and SN032TF04, respectively – were collected from the same snake as the allotype. Morphological data for all paratypes are provided in Supplementary Table S2.

### Diagnosis

Cephalic bulb consists of 2 smooth swellings, 1 dorsal and 1 ventral (Figs 1A, 2A and 3D). Distance from anterior end to cervical sac short (approximately 0.17 for both sexes). Distance from anterior end to excretory pore short (approximately 0.53 for both sexes). Excretory pore, positioned ventrally always anterior to cervical papillae (approximately 0.59 both sexes), which are positioned on lateral sides (Figs 1A and 2A, F). Males exhibit usually 8 or 7 pairs of caudal papillae, comprising 3 preanal, 1 paranal, and 3 or 4 postanal (Figs 1B, 2H and 3B). Females possess 1 caudal papillary pair located near tail end, with each positioned on dorsal and ventral sides, laterally to midlines (Figs 1C, 2I and 3A). Didelphic uterus very long, about 3 of 5 of the body lengths (Fig. 1E).

### Comparison with other *Tanqua* species

The newly described species, *T. siamensis* sp. nov. has strongly distinctive characteristics (see Table S1). Notably, 1 pair of caudal papillae situated dorsally and ventrally close to the end of the female tail of *T. siamensis* sp. nov. is reported for the first time (Figs 1C, 2I and 3A), setting it apart from all other *Tanqua* species.

*Tanqua tiara* (von Linstow, 1879) is characterized by 4 cephalic bulb swellings (Gibbons and Keymer, 1991; Sou, 2020), despite multiple studies and revisions, resulting in variations in characteristic measurements and reports of diverse hosts among monitor lizard species (see Table S1), In contrast, *T. siamensis* sp. nov. has only 2 bulb swellings (Fig. 2A). Moreover, *T. tiara* was described with 4 branches of the uterus, whereas *T. siamensis* sp. nov. has only 2 branches.

*Tanqua geoclemydis* Wang *et al*., 1979, stands out as the sole *Tanqua* species described from a turtle, the Chinese pond turtle, *M. reevesii*, in China. Relative to *T. siamensis* sp. nov., it has smaller dimensions, including body length (♂ 13.40 and ♀ 14.40–15.40 *vs* ♂ 17–40.33 and ♀ 15.33–41.17), oesophagus length (♂ 0.17 and ♀ 0.14–0.18 *vs* ♂ 0.30–0.54 and ♀ 0.21–0.67), the number of head-bulb swellings (4 *vs* 2), appearance of cervical sacs (asymmetrical *vs* symmetrical) and spicule length (0.56 *vs* 1.09–1.87).

*Tanqua occlusa* Schuurmans-Stekhoven, 1943, from the oesophagus and stomach of Smith's African water snake, *G. smithii*, in Congo, Africa, was described with limited information. However, it evidently has 4 cephalic bulb swellings, akin to *T. tiara*. Males also exhibit 5 pairs of caudal papillae (compared with 7 or 8 pairs in *T. siamensis* sp. nov.), and females are large, ranging from 45 to 62 (compared to 15–41) (Schuurmans-Stekhoven, 1943).

*Tanqua gigantica* Kung, 1948, from the intestine of snakes in Southeast Asia, is a very large species with a total body length of 110–130 (*vs* 17–40 in *T. siamensis* sp. nov.) with a body width of 2.0–2.5 (*vs* 0.5–1.1) for males, while females are 120–160 (*vs* 15–41) long with a body width of 2.5–3.2 (*vs* 0.5–1.4) (Kung, 1948). *Tanqua gigantica* is also distinguished by having only 6 caudal papillary pairs, a short tail (0.5% of the body length *vs* ♂ 1.2–3.2% and ♀ 2.5–6.0% in *T. siamensis* sp. nov.), and a short spicule length (1% of the body length *vs* 4–8% in *T. siamensis* sp. nov.).

*Tanqua bainae* Ghadirian, 1968, is another large species with a body length of 85–100 with a body width of 1.6 for males and 105–120 and a body width of 2 for females. These body size ranges show no overlap with *T. siamensis* sp. nov. Furthermore, the spicule length of *T. bainae* accounts for only about 1.5% of the total body length (*vs* 4–8% for *T. siamensis* sp.). Similarly, the uterus length of *T. bainae* has been described as about 2% of the body length (*vs* 40–50%). The site of infection (whole digestive tract *vs* stomach only) and locality (Madagascar *vs* Thailand) reported for this species also suggested that it is distinct from *T. siamensis* sp. nov.

A significant distinction between *T. diadema* von Linstow, 1904 and *T. siamensis* sp. nov., lies, firstly, in the documented locality (Brazil *vs* Thailand) and, secondly, in the site of infection (intestines *vs* stomach). Importantly, *T. diadema* possesses a retractable cephalic bulb and pseudolabia within the cuticular collar, forming a prepuce-like sheath (Baylis and Lane, 1920), features not observed in *T. siamensis* sp. nov. (Figs 1A, 2A and 3D). Due to the retractile bulb of *T. diadema*, it has pseudolabia that, although smaller, are relatively about the size of the cephalic bulb (Baylis and Lane, 1920), while *T. siamensis* sp. nov. displays a cephalic bulb by considerably larger than the pseudolabia. Moreover, the excretory pore in *T. diadema* is situated behind the cervical papillae, which contrasts with the positioning in *T. siamensis* sp. nov., where it lies anterior to the cervical papillae. The uterus length is reportedly short in *T. diadema*, whereas this character is notably long in *T. siamensis* sp. nov (40–50% of the total body length).

*Tanqua anomala* (von Linstow, 1904), reported in Indonesia and Australia, is another well-studied species that has undergone taxonomic revisions multiple times, resulting in varied morphological counts and measurements (see Table 1). It resembles *T. siamensis* sp. nov., in several characteristics but several differentiate these 2 species: (1) The distance from the anterior end to the excretory pore is 0.35–0.66 of the body length for males and 0.34–0.73 for females in *T. siamensis* sp. nov. (approximately 0.53 or 2.0% of the body length for both sexes). In contrast, in *T. anomala* it is more distant, 0.75–0.96 for males and 0.90–1.23 for females (approximately 0.85 and 1.09, or 2.3 and 2.9% of the body length for males and females, respectively, Baylis, 1916; Dewi *et al*., 2008). (2) The arrangement of the excretory pore and cervical papillae appears to differ between the 2 species, as the cervical papillae are anterior to the excretory pore in *T. anomala* (Dewi *et al*., 2008; Al-Moussawi, 2010), whereas in *T. siamensis* sp. nov. they are posterior to it (Figs 1A and 2A, 2F). (3) *Tanqua anomala* has been remarked as having a very short uterus, whereas *T. siamensis* sp. nov. has a long uterus. According to these characters, *T. siamensis* sp. nov. should be nominated as new species in the genus *Tanqua*.

### Variation

After analysing the morphological measurements of all *T. siamensis* sp. nov. specimens, no marked morphological variation among individuals or genders were observed. The 2-dimensional plot of PC1 and PC2 axes showed no distinct separation and a significant overlap between males and females (Fig. S2). This lack of differentiation in PCA was supported by PC1 and PC2 accounting for 71.85% of the total variance together, with a substantial drop in eigenvalue between PC1 (60.07%) and PC2 (11.78%), and also between PC2 and PC3 (5.90%). Consequently, it can be inferred that sexual dimorphism of *T. siamensis* sp. nov. can only be determined based on discrete sex characteristics (such as the uterus and vulva in females, and caudal papillary pairs in males), rather than shared measurement characters between both sexes.

The intensity of *T. siamensis* sp. nov. infections ranged from 8 to 52 worms, with a mean of 23 (Table S2). Some morphological variation was observed among *Tanqua* specimens from individual snakes. The analysis among male specimens revealed 3 clusters which partially overlap (Fig. S2B). A similar result was observed for female worms (Fig. S2C), suggesting a minor host impact on the morphology of the worms.

### Genetic characterization and phylogenetic position

**Figure 4.**
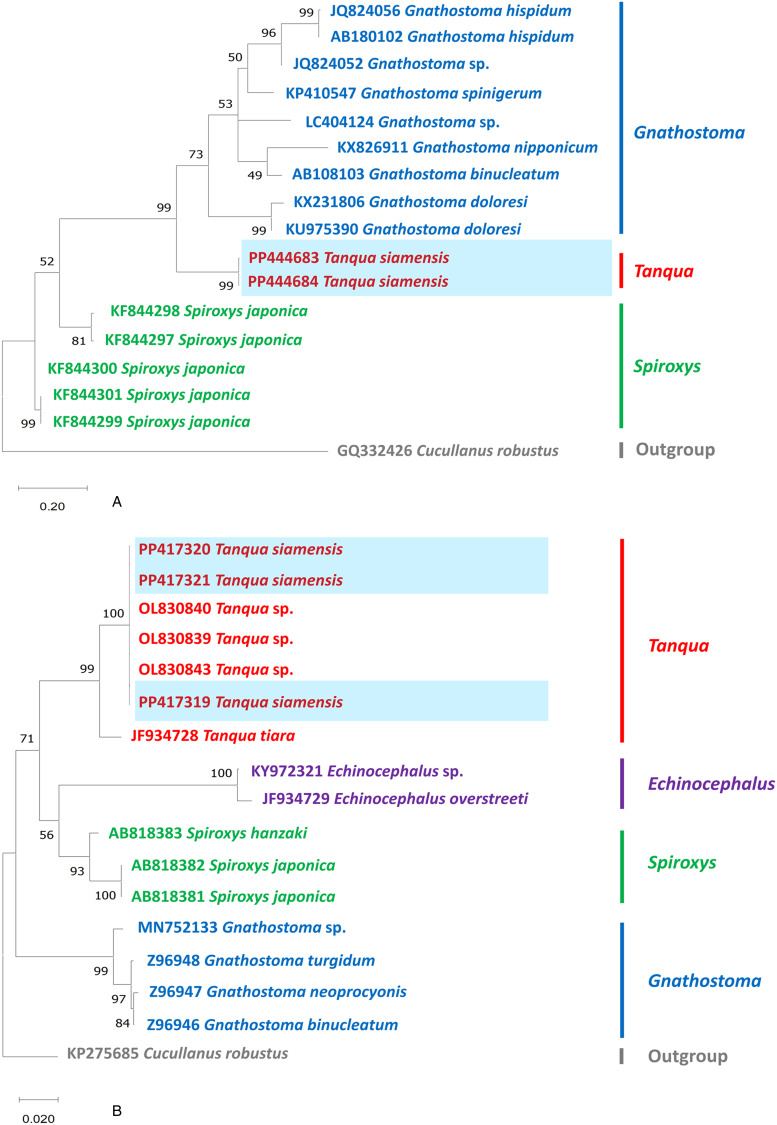
Phylogenetic analysis of the available sequences of nematodes within the family Gnathostomatidae based on different genetic markers: (A) *COI* and (B) 18S rRNA. The analyses were conducted using MEGAX with the maximum likelihood method. Branch length scale bars indicate the number of substitutions per site. Coloured lines/fonts represent genetic data from various genera in Gnathostomatidae, sourced from GenBank, with the red line/font specifically highlighting the genus *Tanqua*. The blue box indicates the specimens of *Tanqua siamensis* sp. nov. utilized in the present study.

Relative to other sequences available for comparison, both phylogenies suggest that *T. siamensis* sp. nov. is a distinct species within Gnathostomatidae. The *COI* analyses suggested that *T. siamensis* sp. nov. forms a distinct clade within Gnathostomatidae (Fig. 4A). Specifically, the nuclear 18S rRNA strongly indicated the differentiation of *T. siamensis* sp. nov. from *T. tiara* and other gnathostome sequences available in GenBank (Fig. 4B). The results illustrated that *T. tiara* is a sister clade to *T. siamensis* sp. nov., confirmed by a 99% Bayesian posterior probability. The genetic results also found that the sequence of *T. siamensis* sp. nov. from Thailand perfectly matches that of the larva of *Tanqua* sp. found in a snakehead fish from Bangladesh (Williams *et al*., 2022), suggesting that they represent the same species. The genetic variation between *T. siamensis* sp. nov. and other reported species ranged from 13 to 18% for the *COI* and 2 to 10% for the 18S rRNA gene. The closest genetic distances for 18S rRNA gene were observed between *T. siamensis* sp. nov. and *T. tiara*, with a 2% difference; for *COI*, a 13% difference was observed with *Gnathostoma binucleatum.*

### Natural history

*Tanqua siamensis* sp. nov., appears to be prevalent in the rainbow water snake, *E. enhydris*, as evidenced by its discovery in all 12 snake specimens examined. This species produces lesions in the stomach of its hosts. In instances of high-level infection, numerous small hardened spots [possibly indicative of caseous necrosis, as revealed by Gibbons and Keymer (1991)] were common on the internal wall of the organ (Fig. S1), where the nematodes firmly affix themselves using the cephalic portion. The parasites densely populate the stomach, resulting in noticeable swelling.

Rainbow water snakes eat small fishes (Cox *et al*., 2012), which serve as intermediate hosts for *T. siamensis* sp. nov. Inspection of the snake gastrointestinal tract unveiled fish carcasses inside the oesophagus and stomach containing worms that had not yet attached to the stomach wall. The role of fish as an intermediate host for transmitting this nematode species is also supported by the phylogenetic results, showing that the genetic sequences of *Tanqua* sp. larvae found in a snakehead fish species, *Channa* sp. (*sensu* Williams *et al*., 2022), matched those of the *Tanqua* specimens in this study. This discovery extends the life cycle and the distribution of *T. siamensis* sp. nov. from South Thailand to Bangladesh, where its adults and larvae were reported, respectively.

## Discussion

Despite the distinctiveness of many *Tanqua* species, several original species descriptions lack sufficient morphological details, particularly *T. gigantica*, *T. bainae*, *T. geoclemydis*, *T. diadema* and *T. occlusal*; each described from a small number of specimens. The first 3 species provided clear morphological measurements such as body length, body width and variables related to the lengths and ratios between reproductive organs and the total body length (Kung, 1948; Ghadirian, 1968), serving to differentiate them from *T. siamensis* sp. nov. Conversely, *T. diadema*, and *T. occlusal* exhibit overlapping ranges of morphological measurements, resulting in difficulties in species differentiation. However, meristic counts and qualitative traits, such as the number of cephalic bulb swellings, the order of cervical papillae and excretory pore, and retractable head capability (Baylis and Lane, 1920; Schuurmans-Stekhoven, 1943), distinguish these and the new species. In the case of *T. tiara* (Gibbons and Keymer, 1991; Agustin *et al*., 2017; Sou, 2020) and *T. anomala* (Baylis, 1916; Johnston and Mawson, 1948; Kagei and Shogaki, 1977; Dewi *et al*., 2008), despite taxonomic revisions with varying numbers of specimens resulting in varied morphological characteristics and wide distribution for these 2 species, there are clear morphological differences that distinguish them from *T. siamensis* sp. nov.

Using the presence of 4 uterine branches as a diagnostic criterion for species identification, as invoked in some previous publications (e.g. Baylis, 1916; Baylis and Lane, 1920; Gibbons and Keymer, 1991), is problematic. Nematodes typically have either 1 or 2 genital tracts – monodelphic or didelphic (Li *et al*., 2017). The observation of 4 tracts in *T. tiara* may be exceptional case, suggesting the need for a more in-depth study of extended anatomy. Alternatively, as uteri are typically folded, the character may have been misinterpreted.

The number of caudal papillary pairs in males is an important character for *Tanqua* species identification (e.g. Baylis and Lane, 1920; Dewi *et al*., 2008; Sou, 2020). However, inconsistencies in reported numbers, particularly for *T. tiara*, pose challenges. Sou (2020) reported 5 pairs, whereas Baylis and Lane (1920) and Gibbons and Keymer (1991) indicated 8 pairs. *Tanqua anomala* also has conflicting reports, with Dewi *et al*. (2008) documenting 8 pairs whereas Johnston and Mawson (1948) and Kagei and Shogaki (1977) reported 5 pairs. The *Tanqua* specimens in this study add some complexity in that they vary in caudal papillae counts, ranging between 7 and (usually) 8 pairs. This variation suggests that caution is warranted when using this character as a diagnostic tool for species identification. Notably, this study is the first to report a caudal papillary pair near the end of the female tail of *T. siamensis* sp. nov. This character is unique to the new species.

The phylogenetic analysis, incorporating both nuclear and mitochondrial genes, indicates that *T. siamensis* sp. nov. is consistent with the genus *Tanqua*, and distinct from *T. tiara*. However, there is little genetic information for *Tanqua* species. Most studies were conducted molecular studies became common. Even well-known species, like *T. anomala*, lack sequences, despite efforts to obtain DNA from previous authors by the researchers in this study (e.g. Dewi *et al*., 2008; Al-Moussawi, 2010). Confirming whether *T. siamensis* sp. nov. is closer to *T. anomala* or *T. diadema* (the most closely resembling in morphology), requires further study involving specimens of these species from within their reported distribution.

The 18S rRNA gene analysis showed that *T. siamensis* sp. nov., is distributed from south Thailand to Bangladesh and that it is transmitted from fishes to snakes. Such a transmission aligns with observed hunting behaviour, which primarily targets fishes (Cox *et al*., 2012), as evidenced by the presence of fish carcasses containing unattached-to-organ *Tanqua* inside the upper digestive tract of the snakes. The reported host range of *T. tiara*, found in monitor lizard species, *Varanus* spp. (e.g. Baylis, 1916; Gibbons and Keymer, 1991; Agustin *et al*., 2017; Sou, 2020), and *T. anomala*, found in semiaquatic snake species (Baylis, 1916; Baylis and Lane, 1920; Johnston and Mawson, 1948; Kagei and Shogaki, 1977; Dewi *et al*., 2008), including the rainbow water snake, *E. endydris*, from Indonesia (Kagei and Shogaki, 1977), suggests the possibility that each *Tanqua* species, including *T. siamensis* sp. nov., may infect multiple hosts.

A few reports have indicated the presence of *T. tiara* and *T. anomala* in snakes in Thailand. Chaiyabutr and Chanhome (2002) documented *T. tiara* in the Laotian wolf snake, *Lycodon laoensis*. However, the details and specimen numbers for both hosts (*n* = 2) and *Tanqua* (*n* = 1) were insufficient for precise species characterization. A similar lack of information was observed in the discovery of *T. anomala* by Baylis and Lane (1920) in the puff-faced water snake, *Homalopsis buccata*. These publications rely solely on basic parasite morphology for species identification. It is also plausible that both *T. tiara* and *T. anomala* exist in Thailand but are specifically hosted by other reptiles.

Sexual dimorphism in *T. siamensis* sp. nov. is challenging to discern solely through general morphological measurements. Host may impact worm morphology, as indicated by distinct clusters in PCA results. However, the low sample size per host suggests the need for more specimens to better understand host-related variation. Studies on nematode sexual dimorphism can be further implemented, such as host environmental influences (e.g. Anjam *et al*., 2020), developmental molecular events (e.g. Emmons, 2014; Pollo *et al*., 2023) and evolutionary perspectives (e.g. Morand and Hugot, 1998; Ancell and Pires-daSilva, 2017). Similar observations have been reported in nematodes infecting snakes, such as *Paracapillaria najae* (Charoennitiwat *et al*., 2023) and *Paracapillaria siamensis* (Charoennitiwat *et al*., 2024), although they are distinct taxa.

In conclusion, both morphological and genetic characterizations provide compelling evidence supporting the identification of a new species within the genus *Tanqua*. This discovery leads to the formal naming of the species as *T. siamensis* sp. nov. However, uncertainties in morphological observations from previous, often older studies raise questions about the reliability of using certain characters for *Tanqua* species identification. The limited popularity of this nematode genus within the scientific community has contributed to a delayed development in all aspects of basic information (e.g. molecular genetics), particularly when compared to medically relevant nematodes. Nonetheless, investigating *T. siamensis* sp. nov. in this study has not only expanded the taxonomy of its genus but also raised awareness of parasitic infections and lesions, especially for captive snakes.

## Supporting information

Charoennitiwat et al. supplementary material 1 Charoennitiwat et al. supplementary material

Charoennitiwat et al. supplementary material 2 Charoennitiwat et al. supplementary material

Charoennitiwat et al. supplementary material 3 Charoennitiwat et al. supplementary material

Charoennitiwat et al. supplementary material 4 Charoennitiwat et al. supplementary material

## Data Availability

The data that support the findings of this study are available from the first and corresponding authors upon reasonable request.

## Supplementary material

The supplementary material for this article can be found at https://doi.org/10.1017/S0031182024000908.

## References

[ref1] Agustin ALD, Koesdarto S, Lukiswanto BS, Suwanti LT, Arifin Z and Putranto ED (2017) Morphological identification nematodes *Tanqua tiara* found on gastric *Varanus salvator* at East Java. KnE Life Sciences 3, 668.

[ref2] Al-Moussawi AA (2010) First record in Iraq of *Tanqua anomala* (Linstow, 1904) from the dice snake, *Natrix tessellata tessellata* (Laurenti, 1768). Bulletin of the Iraq Natural History Museum 11, 27–38.

[ref3] Ancell H and Pires-daSilva A (2017) Sex-specific lifespan and its evolution in nematodes. Seminars in Cell & Developmental Biology 70, 122–129. 28554570 10.1016/j.semcdb.2017.05.012

[ref4] Anjam MS, Shah SJ, Matera C, Różańska E, Sobczak M, Siddique S and Grundler FMW (2020) Host factors influence the sex of nematodes parasitizing roots of *Arabidopsis thaliana*. Plant, Cell & Environment 43, 1160–1174. 10.1111/pce.13728 32103526

[ref5] Baylis HA (1916) XIX. – the nematode genus *Tanqua*, R. Blanchard. Annals and Magazine of Natural History 17, 223–232.

[ref6] Baylis HA and Lane, C (1920) A revision of the nematode family Gnathostomidae. Proceedings of the Zoological Society of London 90, 245–310.

[ref7] Bilqees FM (1980) A note on *Tanqua anamala* (Linstow, 1904) (syn. *Tanqua sindensis* Farooq *et al*., 1979). Pakistan Journal of Zoology 12, 268.

[ref8] Blanchard R (1904) *Tanqua*, n. g., remplaçant *Ctenocephalus* von Linstow. Archives de Parasitologie 8, 478.

[ref9] Chaiyabutr N and Chanhome L (2002) Parasites in snakes of Thailand. Bulletin of the Maryland Herpetological Society 38, 39–50.

[ref10] Chan AHE, Chaisiri K, Dusitsittipon S, Jakkul W, Charoennitiwat V, Komalamisra C and Thaenkham U (2020) Mitochondrial ribosomal genes as novel genetic markers for discrimination of closely related species in the *Angiostrongylus cantonensis* lineage. Acta Tropica 211, 105645. 32702297 10.1016/j.actatropica.2020.105645

[ref11] Charoennitiwat V, Chaisiri K, Ampawong S, Laoungbua P, Chanhome L, Vasaruchapong T, Tawan T, Thaenkham U and Ratnarathorn N (2023) Redescription and new record of *Paracapillaria* (*Ophidiocapillaria*) *najae* (Nematoda: Trichuroidea) in the monocled cobra *Naja kaouthia* from central Thailand: morphological and molecular insights. Parasitology 150, 901–910. 37519244 10.1017/S0031182023000707 PMC10577661

[ref12] Charoennitiwat V, Chaisiri K, Kanjanapruthipong T, Ampawong S, Chanhome L, Vasaruchapong T, Thaenkham U and Ratnarathorn N (2024) *Paracapillaria* (*Ophidiocapillaria*) *siamensis* sp. nov. (Nematoda: Trichuroidea): a new nematode in *Naja kaouthia* from Thailand. Parasitology 151, 529–538. 38659195 10.1017/S0031182024000404 PMC11106506

[ref13] Cox MJ, Hoover M, Chanhome L and Thirakhupt K (2012) The Snakes of Thailand. Bangkok: Chulalongkorn University Museum of Natural History.

[ref14] Dewi K, Jones H and Hamidy A (2008) The status of *Tanqua anomala* (Von Linstow, 1904 (Nematoda: Gnathostomatoidea). Transactions of the Royal Society of South Australia 132, 7–13.

[ref15] Eamsobhana P, Lim PE and Yong HS (2015) Phylogenetics and systematics of *Angiostrongylus* lungworms and related taxa (Nematoda: Metastrongyloidea) inferred from the nuclear small subunit (SSU) ribosomal DNA sequences. Journal of Helminthology 89, 317–325. 24622302 10.1017/S0022149X14000108

[ref16] Emmons SW (2014) The development of sexual dimorphism: studies of the *Caenorhabditis elegans* male. Wiley Interdisciplinary Reviews. Developmental Biology 3, 239–262. 25262817 10.1002/wdev.136 PMC4181595

[ref17] Farooq M, Khanum Z and Zuberi HB (1979) A new nematode *Tanqua sindensis* (Nematoda; Gnathostomatidae) from freshwater snake of Kalri Lake, Sind, Pakistan. Pakistan Journal of Zoology 11, 163–165.

[ref18] Ghadirian E (1968) Nématodes parasites d'Ophidiens Malgaches. Mémoires du Museum National d'Histoire Naturelle 54, 1–54.

[ref19] Gibbons LM and Keymer IF (1991) Redescription of *Tanqua tiara* (Nematoda, Gnathostomidae), and associated lesions in the stomach of the Nile monitor lizard (*Varanus niloticus*). Zoologica Scripta 20, 7–14.

[ref20] Hall TA (1999) Bioedit: a user-friendly biological sequence alignment editor and analysis program for Window 98/98/NT. Nucleic Acids Symposium Series 41, 95–98.

[ref21] Hammer O, Harper D and Ryan P (2001) PAST: paleontological statistics software package for education and data analysis. Palaeontologia Electronica 4, 1–9.

[ref22] Holterman M, van der Wurff A, van den Elsen S, van Megen H, Bongers T, Holovachov O, Bakker J and Helder J (2006) Phylum-wide analysis of SSU rDNA reveals deep phylogenetic relationships among nematodes and accelerated evolution toward crown clades. Molecular Biology and Evolution 23, 1792–1800. 16790472 10.1093/molbev/msl044

[ref23] Johnston TH and Mawson PM (1948) Some new records of nematodes from Australian snakes. Records of the South Australian Museum 9, 101–106.

[ref24] Kagei N and Shogaki Y (1977) Helminths of animals imported to Japan. Japanese Journal of Tropical Medicine and Hygiene 5, 155–159.

[ref25] Kung CC (1948) On some new species of spirurids from terrestrial vertebrates, with notes on *Habronema mansioni*, *Physaloptera paradoxa* and *Hartertia zuluensis*. Journal of Helminthology 22, 141–164.

[ref26] Laetsch DR, Heitlinger EG, Taraschewski H, Nadler SA and Blaxter ML (2012) The phylogenetics of Anguillicolidae (Nematoda: Anguillicoloidea), swimbladder parasites of eels. BMC Evolutionary Biology 12, 60. 22559142 10.1186/1471-2148-12-60 PMC3503875

[ref27] Li Q, Liang W, Zhang X and Mahamood M (2017) Nematode genera and species description along the transect. In Soil Nematodes of Grasslands in Northern China. Hangzhou, China: Academic Press, pp. 45–228. doi: 10.1016/B978-0-12-813274-6.00003-X.

[ref28] Lopez LK and Duffy MA (2021) Mechanisms by which predators mediate host–parasite interactions in aquatic systems. Trends in Parasitology 37, 890–906. 34281798 10.1016/j.pt.2021.06.006

[ref29] Morand S and Hugot JP (1998) Sexual size dimorphism in parasitic oxyurid nematodes, Biological Journal of the Linnean Society 64, 397–410.

[ref30] Pollo SMJ, Leon-Coria A, Liu H, Cruces-Gonzalez D, Finney CAM and Wasmuth JD (2023) Transcriptional patterns of sexual dimorphism and in host developmental programs in the model parasitic nematode *Heligmosomoides bakeri*. Parasites & Vectors 16, 171. 37246221 10.1186/s13071-023-05785-2 PMC10225086

[ref31] Schneider JG (1799) Historiae Amphibiorum Narturalis et Literariae. Fasciculus Primus, Continens Ranas. Calamitas, Bufones, Salamandras et Hydros. Jena: Frommanni.

[ref32] Schuurmans-Stekhoven JH (1943) Parasitic nematodes from the Belgian Congo. Bulletin du Musée Royale d'Histoire Naturelle de Belgique 19, 1–20.

[ref33] Sou SK (2020) Redescription of Tanqua tiara (von Linstow, 1879) Blanchard, 1904 (Nematoda: Gnathostomatidae) from *Varanus flavescens* (Hardwicke and Gray, 1827) (Reptilia: Varanidae) from Birbhum district, West Bengal, India. Journal of Parasitic Diseases 44, 381–387. 32508412 10.1007/s12639-020-01197-6 PMC7244700

[ref34] Tamura K, Stecher G, Peterson D, Filipski A and Kumar S (2013) MEGA6: molecular evolutionary genetics analysis version 6.0. Molecular Biology and Evolution 30, 2725–2729. 24132122 10.1093/molbev/mst197 PMC3840312

[ref35] Terrell PS and Stacy AB (2007) Infectious Diseases and Pathology of Reptiles. Boca Raton: CRC Press.

[ref36] Thaenkham U, Chaisiri K and Chan AHE (2022) Molecular Systematics of Parasitic Helminths. Singapore: Springer.

[ref37] Thompson J, Gibson T and Higgins D (2002) Multiple sequence alignment using ClustalW and ClustalX. Current Protocols in Bioinformatics, Chapter 2, Unit 2.3. doi: 10.1002/0471250953 18792934

[ref38] Tokiwa T, Harunari T, Tanikawa T, Komatsu N, Koizumi N, Tung KC, Suzuki J, Kadosaka T, Takada N, Kumagai T and Akao N (2012) Phylogenetic relationships of rat lungworm, *Angiostrongylus cantonensis*, isolated from different geographical regions revealed widespread multiple lineages. Parasitology International 61, 431–436. 22387862 10.1016/j.parint.2012.02.005

[ref39] Vattakaven T, George R, Balasubramanian D, Réjou-Méchain M, Muthusankar G, Ramesh B and Prabhakar R (2016) India biodiversity portal: an integrated, interactive and participatory biodiversity informatics platform. Biodiversity Data Journal 4, e10279. 10.3897/BDJ.4.e10279 PMC5136667 27932923

[ref40] von Linstow OFB (1879) Helminthologische untersuchungen. Jahreshefte des Vereins für Vaterländische Naturkunde in Württemberg 35, 313–342.

[ref41] von Linstow OFB (1904) Nematoda in the collection of the Colombo Museum. Spolia Zeylanica 1, 91–104.

[ref42] Wang PQ, Zhao YR, Wang XY and Zhang JY (1979) Report on some nematodes from vertebrates in Central and South China. Fujian Shida Xuebao 2, 78–92.

[ref43] Williams M, Hernandez-Jover M, Hossen MS and Shamsi S (2022) Genetic characterisation of *Tanqua* (von Linstow, 1879) (Nematoda: Gnathostomatidae) larval forms including new host and locality records. International Journal for Parasitology: Parasites and Wildlife 17, 127–132. 35059288 10.1016/j.ijppaw.2022.01.001 PMC8760434

