# Supplementary material for: A new nematode species, Tanqua siamensis sp. nov. (Nematoda: Gnathostomatidae) in the rainbow water snake, Enhydris enhydris, from Thailand
Source: Parasitology. 2024 Sep 23;151(8):821–31. doi: 10.1017/S0031182024000908 (PMC11579038; doi:10.1017/S0031182024000908)
Supplement: Charoennitiwat et al. supplementary material 1 — Charoennitiwat et al. supplementary material [file S0031182024000908sup001.pdf]

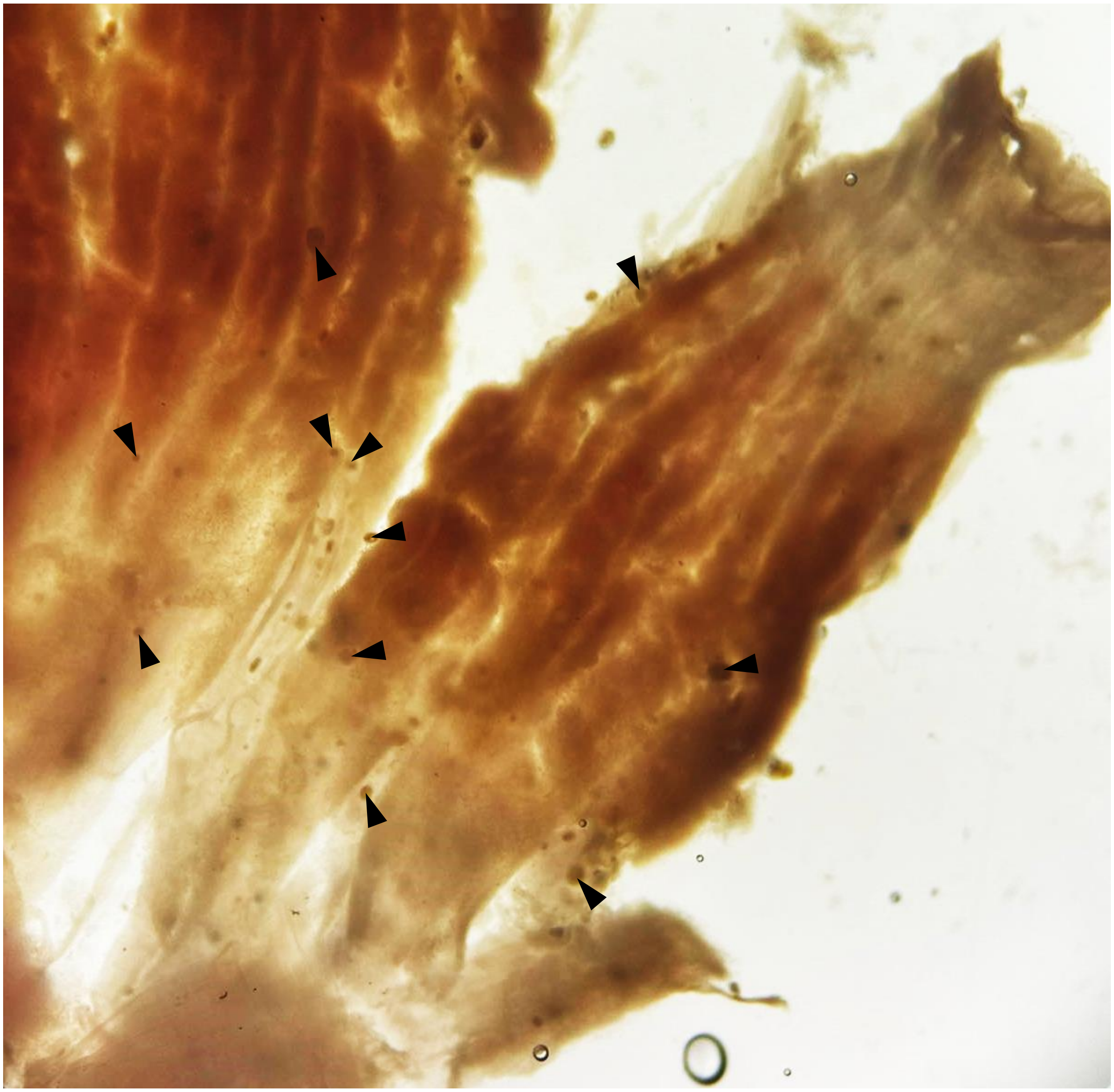

Figure S1: Lesions manifest prominently in cases of high infection with *Tanqua siamensis* sp. nov. The small, hardened spots (caseous necrosis) are dispersed throughout the internal wall of the snake's stomach (black arrows).
