# Supplementary material for: A new nematode species, Tanqua siamensis sp. nov. (Nematoda: Gnathostomatidae) in the rainbow water snake, Enhydris enhydris, from Thailand
Source: Parasitology. 2024 Sep 23;151(8):821–31. doi: 10.1017/S0031182024000908 (PMC11579038; doi:10.1017/S0031182024000908)
Supplement: Charoennitiwat et al. supplementary material 2 — Charoennitiwat et al. supplementary material [file S0031182024000908sup002.pdf]

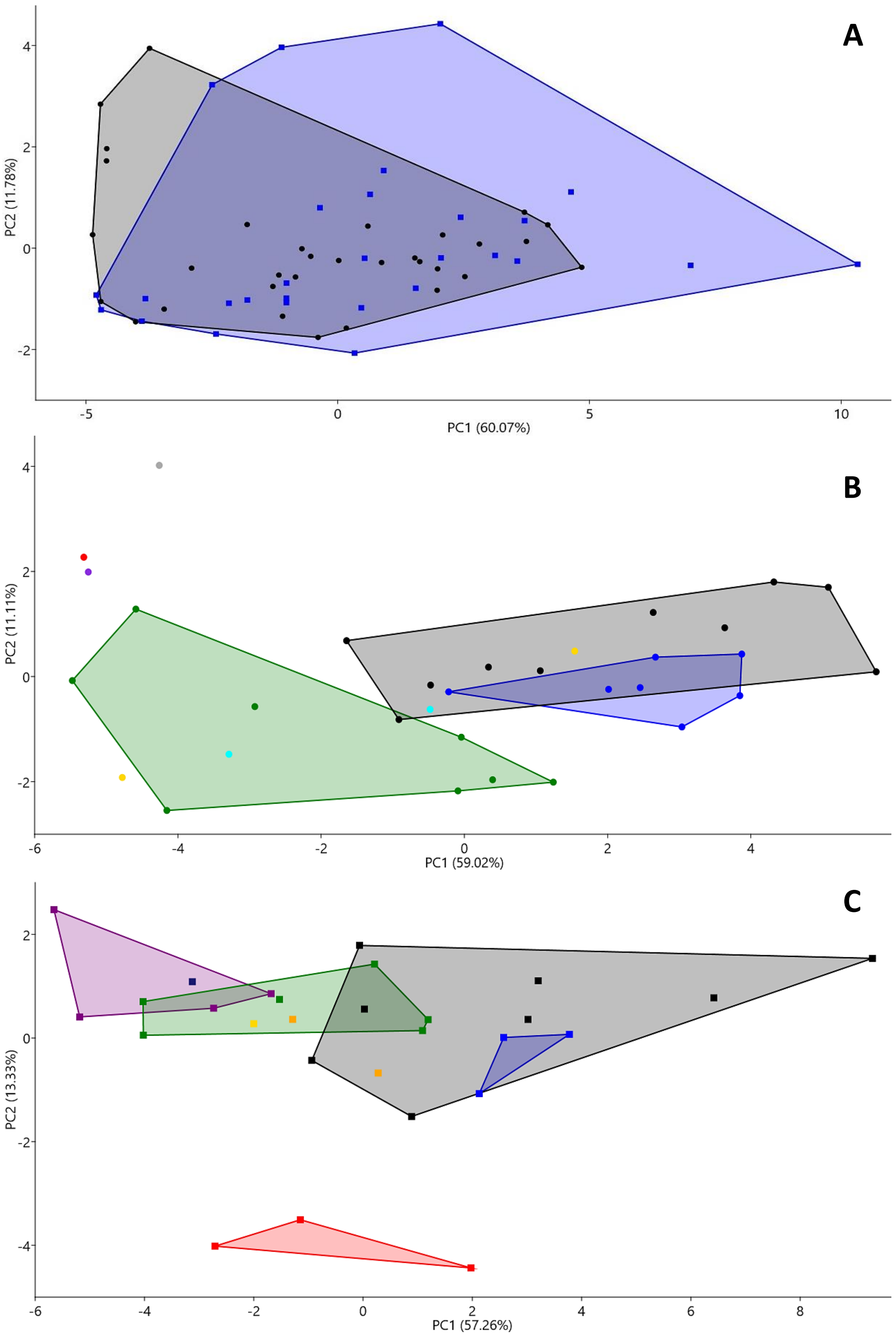

A
PC2 (11.78%)
PC1 (60.07%)
B
PC2 (11.11%)
PC1 (59.02%)
C
PC2 (13.33%)
PC1 (57.26%)

Figure S2: Principal component analysis of *Tanqua siamensis* sp. nov. specimens: (A) PCA of all samples based on 17 examined morphological characters for both genders (71.85% of total variance). Black dots and lines represent individual male specimens and their group, respectively, while blue squares and lines represent individual female specimens and their group; (B) PCA of all male specimens based on 19 examined morphological characters (70.13% of total variance). Coloured dots represent individual male *T. siamensis* sp. nov. specimens hosted in different snake specimens, and corresponding-coloured lines reveal their clustering; (C) PCA of all female specimens based on 20 examined morphological characters (70.59% of total variance). Coloured dots represent individual female *T. siamensis* sp. nov. specimens hosted in different snake specimens, and corresponding-coloured lines reveal their clustering.
